# Treatment of Facial Contour and Localized Submental Fat Using Multilayer Microneedling Radiofrequency

**DOI:** 10.1111/jocd.71160

**Published:** 2026-09-29

**Authors:** Xavier Eduardo Garzon Garzon, Gustavo Robertson Filippo, Bibiana Florian da Costa, Paula Regazzi de Gusmão, Alexandre de Almeida Filippo

**Affiliations:** ^1^ Institute of Dermatology Professor Rubem David Azulay Rio de Janeiro Brazil

**Keywords:** facial aging, facial contouring, microneedling radiofrequency, skin laxity, skin rejuvenation, submental fat

## Abstract

**Background:**

Microneedling radiofrequency has gained prominence in the treatment of cervical skin laxity and localized submental fat, offering promising aesthetic outcomes; however, further clinical evidence is needed to support its efficacy and safety.

**Aim:**

To evaluate the efficacy and safety of the Megaderme Duo platform in improving facial contour and reducing submental fat.

**Methods:**

Sixteen participants (12 women and 4 men) with localized submental fat classified as Grades 1–4 according to the PASMFRS underwent three microneedling radiofrequency sessions at four‐week intervals using the Megaderme Duo RF Multi‐Layer Eletroderme XL LMG platform. Dermal, subcutaneous, and subplatysmal fat thickness were assessed using high‐frequency Doppler ultrasound. Statistical analyses were performed using SPSS software.

**Results:**

Statistically significant reductions were observed in subcutaneous tissue thickness (*p* = 0.001) and subplatysmal fat thickness (*p* = 0.034). A significant bilateral reduction in subcutaneous thickness at the mandibular angles was also detected (*p* < 0.001). Comparative analysis of pre‐ and post‐treatment photographs using the PASMFRS revealed significant improvement (*p* = 0.001), with 68.75% of participants showing a reduction of at least one grade. Participant satisfaction was high, with 93.8% reporting favorable outcomes. No severe or difficult‐to‐manage adverse effects were observed.

**Conclusion:**

Multilayer microneedling radiofrequency using the Megaderme Duo platform proved to be effective in improving facial contour and reducing submental fat, demonstrating a favorable safety profile and high participant satisfaction.

## 1 Introduction

Facial aging is a complex process involving changes in the skin, underlying fat compartments, and skeletal structure. The submental region and the jawline are particularly susceptible to signs of aging, such as skin laxity, fat accumulation, and loss of definition [1, 2, 3]. These changes may have psychological, emotional, and social impacts, as facial aging can affect self‐perception and the way individuals are perceived by others [4].

Currently, skin rejuvenation using radiofrequency (RF) has been widely adopted due to its safety and efficacy. Procedures employing fractional RF, with either insulated or non‐insulated microneedles, create controlled skin penetrations that induce partial thermal damage to the epidermis, followed by fibrotic remodeling [4]. In addition, RF has been shown to initiate re‐epithelialization and to promote dermal coagulation columns characterized by mixed inflammatory infiltration, angiogenesis, and granulation tissue formation within the first days after treatment [5, 6].

Other studies have demonstrated that RF microneedling produces localized and focused dermal injury while sparing the epidermis, thereby reducing the risk of undesirable erythema, pigmentary alterations, and scarring when compared with traditional laser treatments [5, 6]. This technique aims to enhance aesthetic outcomes by decreasing scarring burden and visible signs of aging, such as rhytides [7]. RF microneedling techniques can be successfully applied in patients with a wide range of skin tones and types, particularly those with darker skin, and the safety and efficacy of these treatments have been well established [7, 8, 9, 10].

A prospective study evaluated nine participants with a mean age of 47.6 years (Fitzpatrick skin phototypes II–IV; baseline FEWS score between 3 and 6) who underwent six sessions of fractional radiofrequency treatment using ultrafine electrodes measuring 1.0 mm in length and 0.15 mm in diameter, designed to improve facial skin texture and reduce wrinkles. Three months after the final session, a significant reduction in FEWS scores was observed, from 4.33 ± 0.67 to 3.33 ± 0.67 (*p* < 0.005) [11]. In addition, 88.9% of participants demonstrated global improvement according to the Global Aesthetic Improvement Scale (GAIS), and all reported improved skin texture and wrinkle attenuation. The treatment was well tolerated, with no adverse events and no downtime required. The study also included histological analysis of porcine skin treated with different energy levels (low, medium, and high), demonstrating a fractional pattern of epidermal ablation and dermal coagulation interspersed with areas of preserved tissue, followed by progressive re‐epithelialization over 13 days. Taken together, these findings indicate that fractional radiofrequency technology with ultrafine electrodes is effective in improving skin texture and reducing wrinkles without interfering with participants' daily activities [11, 12].

Despite these preliminary studies, further research is needed on radiofrequency technologies combined with microneedling, which have become increasingly popular due to their accessibility and promising aesthetic outcomes. In this context, the present study evaluated the efficacy and safety of the Megaderme Duo platform in improving facial contour and the submental region, aiming to establish appropriate and comfortable treatment parameters that minimize adverse effects and optimize clinical outcomes.

## 2 Materials and Methods

### 2.1 Study Design

This was an experimental, longitudinal, prospective, single‐center study conducted at the Professor Rubem David Azulay Institute of Dermatology, Laser Department, between September 2024 and February 2025. The investigation was carried out using multilayer microneedling radiofrequency technology with the Megaderme Duo LMG platform.

The study evaluated the efficacy of microneedling radiofrequency, a minimally invasive technology that combines microneedles with thermal energy to promote remodeling of different layers of the skin and subcutaneous tissue. The objective was to analyze its impact on skin laxity of the mandibular contour and submental fat by comparing data obtained before treatment initiation and after completion of the treatment sessions.

### 2.2 Patients

Personal and demographic data were collected during the initial consultation interview, including personal and family medical history, previous surgical and non‐surgical aesthetic procedures, use of continuous medications, and known allergies. All information was recorded in the Meddit electronic medical record system. A clinical evaluation was subsequently performed to identify the presence of skin laxity in the mandibular contour and submental fat, the latter being classified according to the Physician‐Assisted Submental Fat Rating Scale (PASMFRS). At the end of the visit, patients who met the inclusion and exclusion criteria were invited to participate in the study. After completion of the screening process, 16 patients were enrolled.

Inclusion criteria comprised patients aged 18 years or older, regardless of sex, gender identity, race, ethnicity, or skin phototype. Eligible participants included individuals with controlled collagen‐related diseases, localized submental fat classified as Grades 1 to 4 according to the PASMFRS, and skin laxity in the lower third of the face, as well as the absence of prior surgical or non‐surgical treatments in the submental area within the 6 months preceding the first consultation. Exclusion criteria included patients under 18 years of age; users of oral anticoagulants or antiplatelet agents; individuals with pacemakers; those with uncontrolled collagen diseases; pregnant or breastfeeding women; individuals who had undergone surgical procedures or energy‐based device treatments in the neck region within the previous 6 months; those who had received fat‐dissolving substances in the treatment area during the same period; and patients with signs or presence of active infection on the face or in the treated regions.

### 2.3 Treatment Protocol

After participant enrollment, three treatment sessions were performed at four‐week intervals. The procedures followed the protocol outlined below:

- Participants attended consultations at the Laser Department of the Professor Rubem David Azulay Institute of Dermatology, where medical history and physical examination were conducted using the Physician‐Assisted Submental Fat Rating Scale (PASMFRS).
- All information was recorded in the Meddit electronic medical record system.
- Three treatment sessions were prescribed, with a 30‐day interval between each session (four‐week intervals).
- Participants completed the Informed Consent Form (ICF).
- Participants signed the Authorization for Use of Data and Images.
- Standardized photographs were obtained in multiple positions (frontal view, bilateral profile, bilateral 45° oblique views, head flexed with the chin touching the chest, with and without muscle contraction), all taken at a distance of 1.5 m.
- Dermal thickness and submental and mandibular fat thickness were measured using high‐frequency dermatologic ultrasound with Doppler imaging at the Ultrasound Department of the IDPRDA. Measurements were performed using an external probe (Micro Linear Transducer L8‐18i‐RS/2024 GE HealthCare) before treatment, always by the same radiologist, at three standardized anatomical points.
- On the day of the procedure, topical anesthesia with 4% lidocaine (Dermomax) was applied to the submental region and mandibular contour at least 60 min prior to treatment. In addition, Deocil SL (Ketorolac Tromethamine) 10 mg was administered sublingually—one tablet 1 h before and another 15 min before the procedure.
- Patients with a previous history of herpes simplex infection received antiviral prophylaxis with acyclovir 200 mg every 4 h for 5 days.
- The treatment area was cleansed with alcoholic chlorhexidine using sterile gauze.
- Treatment areas were marked with a dermatologic marker, taking care to avoid the marginal mandibular nerve region.

### 2.4 Submental Area Treatment

A line was delineated 3 cm below the mandibular border. From the lateral borders of the sternocleidomastoid muscles, a 2 cm line was drawn medially. The inferior limit of the marked area corresponded to the superior border of the hyoid bone (Figure 1).

**FIGURE 1:**
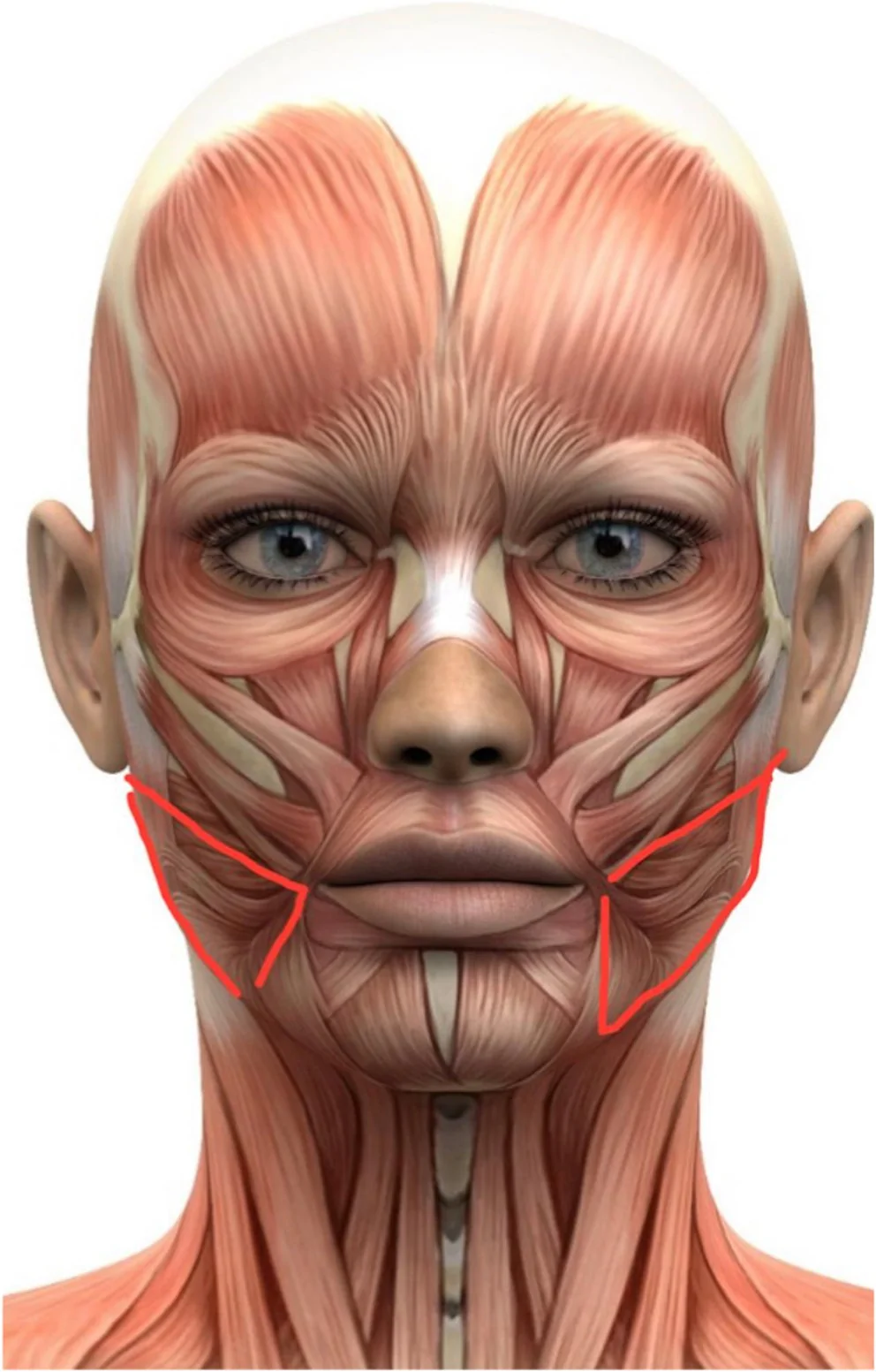
Schematic representation of the treatment area for mandibular contouring. The red outlines indicate the bilateral regions treated along the jawline and adjacent submandibular area.

### 2.5 Mandibular Contour Area

- A vertical line was drawn 1 cm anterior to the vertical ramus of the mandible and the preauricular region, followed by a second parallel line drawn 2 cm from the first.
- A line was delineated 3 cm above the mandibular border, with an additional parallel line drawn 2 cm from the previously marked line.
- At 2 cm posterior to the oral commissure, an additional boundary line was drawn, respecting the previously established inferior limit.

### 2.6 Use of the Platform

- The Megaderme Duo RF MULTI LAYER ELETRODERME XL LMG platform was used for all procedures.
- A 40–gold‐coated microneedle tip was employed, performing three passes at varying depths of 2, 4, and 6 mm, with higher energy levels (15–30 W) and 100 ms pulse duration. The radiofrequency device was applied at a 90° angle relative to the skin surface.
- At the end of the procedure, the treated area was cleansed with alcoholic chlorhexidine or 70% alcohol.
- Daily use of broad‐spectrum sunscreen (SPF 50+) was recommended, with reapplication every 4 h, in addition to the use of a skin‐repairing cream.
- Patients returned at 30‐ and 60‐day intervals for repetition of the treatment protocol.
- At the 90‐day follow‐up visit, new standardized photographs and high‐frequency dermatologic ultrasound with Doppler examinations were performed to measure dermal thickness and fat thickness in the mandibular contour and submental regions.
- After completion of the three sessions, patients completed questionnaires assessing pain using a visual analog scale, treatment effects, and overall satisfaction (perception).

### 2.7 Dermatologic Ultrasound and Treatment Records

Measurements of the different layers of the treated areas were assessed before and after treatment using high‐frequency dermatologic ultrasound with 18–22 MHz probes. The analyzed variables included subcutaneous thickness (from the platysma to the dermis, including preplatysmal fat), submental dermal thickness, and deep subplatysmal fat thickness. In the mandibular region, bilateral subcutaneous thickness and dermal thickness were evaluated.

In addition, standardized photographic documentation was obtained before and after treatment to determine the visual effects following completion of the entire therapeutic protocol. Each patient completed structured questionnaires designed to assess the degree of satisfaction with the procedures and any post‐procedural effects experienced.

### 2.8 Statistical Analysis

Statistical analysis was performed using SPSS software, version 28.0. Numerical variables were described as mean and standard deviation, and categorical variables as absolute and relative frequencies. Comparisons of measurements before and after the intervention were performed using the paired Student's *t*‐test. PASMFRS scores were compared between time points using the Wilcoxon test. A significance level of 5% was adopted (*p* < 0.05).

### 2.9 Ethical Principles

The present study complied with all requirements of Resolution No. 466, dated December 12, 2012, which establishes guidelines and regulatory standards for research involving human subjects, and Resolution No. 510, dated April 7, 2016, which addresses standards applicable to research in the Human and Social Sciences.

Study participants were approached during clinical consultations at the Laser, Cosmiatry, and Private Outpatient Clinics of the Professor Rubem David Azulay Institute of Dermatology, where they were informed about the data collection procedures, treatment protocol, number of sessions, as well as the potential benefits, discomforts, and risks involved in the study.

All individuals who expressed interest in participating, met the inclusion criteria, and agreed to participate freely and voluntarily signed the Informed Consent Form (ICF) and the Authorization for Use of Data and Images. Participants were informed that they were free to decline participation or withdraw consent at any time without any form of penalty. Patients who participated in and completed the entire study protocol incurred no costs and received no financial compensation or benefits related to their participation.

## 3 Results

### 3.1 Patient Profile

The study included 16 participants with a mean age of 39.2 years (±11.6), of whom 75% were female (Table 1).

**TABLE 1:** Demographic characteristics of the study sample according to age and sex.

Variables	*n* = 16
Age (years)—mean ± SD	39.2 ± 11.6
Sex—*n* (%)	
Male	4 (25.0)
Female	12 (75.0)

### 3.2 Submental Area

After treatment of the submental area, dermal thickness increased from 1.6 ± 0.3 mm to 1.7 ± 0.4 mm, with no statistically significant difference (*p* = 0.323), suggesting that the intervention did not affect this structural skin layer. Preplatysmal subcutaneous tissue thickness decreased significantly from 5.5 ± 1.5 mm to 4.4 ± 1.1 mm (*p* = 0.001), demonstrating the effectiveness of the procedure in this region. Deep subplatysmal fat thickness showed a moderately significant reduction, from 5.3 ± 1.7 mm to 4.8 ± 1.9 mm (*p* = 0.034), indicating a favorable effect of the intervention in this area.

### 3.3 Right Mandibular Contour

In the right mandibular contour area, pre‐intervention dermal thickness was 1.6 ± 0.2 mm, increasing to 1.7 ± 0.4 mm after treatment, with no significant difference (*p* = 0.598). Regarding subcutaneous thickness of the right mandibular contour, a significant reduction was observed, from 4.4 ± 1.3 mm to 3.4 ± 1.0 mm (*p* = 0.001).

### 3.4 Left Mandibular Contour

Dermal thickness in the left mandibular contour region was 1.7 ± 0.2 mm before the intervention and remained unchanged at 1.7 ± 0.2 mm after the procedure, with no statistically significant difference (*p* = 0.343). Subcutaneous thickness of the left mandibular contour decreased significantly from 4.5 ± 1.0 mm to 3.6 ± 1.0 mm (*p* = 0.001) following the intervention (Table 2).

**TABLE 2:** Comparison of dermatologic ultrasound parameters before and after the intervention in the submental and mandibular contour regions.

Variables	Before	After	*p*
	Média ± DP	Média ± DP	
**Submental**			
Dermal thickness (mm)	1.6 ± 0.3	1.5 ± 0.2	0,323
Subcutaneous thickness (from the platysma to the dermis) (mm)	5.5 ± 1.5	4.4 ± 1.1	**0.001**
Deep subplatysmal fat thickness (mm)	5.3 ± 1.7	4.8 ± 1.9	**0.034**
**Right Mandibular Angle**			
Dermal thickness (mm)	1.6 ± 0.2	1.7 ± 0.4	0.598
Subcutaneous thickness (mm)	4.4 ± 1.3	3.4 ± 1.0	**< 0.001**
**Left Mandibular Angle**			
Dermal thickness (mm)	1.7 ± 0.1	1.7 ± 0.2	0.43
Subcutaneous thickness (mm)	4.5 ± 1.0	3.6 ± 1.0	**< 0.001**

*Note:* Bold values indicate statistically significant differences (*p* < 0.05).

### 3.5 Satisfaction With Results

Based on patient satisfaction questionnaires and self‐reported perceptions, a high level of acceptance and satisfaction with the treatment outcomes was observed. Specifically, 43.8% (7 patients) reported being extremely satisfied with the results, 50% (8 patients) reported being satisfied, and only 6.3% (1 patient) reported being indifferent, perceiving neither improvement nor worsening after the intervention (Table 3).

**TABLE 3:** Patient‐reported satisfaction with the microneedling radiofrequency intervention.

Variables	*n* = 16
**Satisfaction level—*n* (%)**	
Indifferent	1 (6.3)
Satisfied	8 (50.0)
Extremely satisfied	7 (43.8)

### 3.6 Pain Intensity

During the intervention, which uses microneedles to deliver energy into deeper skin layers, the level of pain reported was predominantly moderate, according to the Visual Analog Scale (VAS). The mean VAS score was 6.5 ± 1.2. Severe pain (VAS 7–10) was reported by 37.5% (6 patients), while moderate pain (VAS 4–6) was reported by 62.5% (10 patients). No participants reported mild pain or absence of pain, indicating that the procedure was associated with considerable discomfort (Table 4).

**TABLE 4:** Pain intensity assessed using the Visual Analog Scale (VAS) during the treatment sessions.

VAS score—mean ± SD	6.5 ± 1.2
**VAS category—*n* (%)**	
No pain (0)	0 (0.0)
Mild (1–3)	0 (0.0)
Moderate (4–6)	10 (62.5)
Severe (7–10)	6 (37.5)

### 3.7 Patient‐Reported Effects

Microneedling radiofrequency is a minimally invasive procedure, and most participants (75%) experienced at least one side effect, with some reporting more than one. The most frequently observed effect was erythema (62.5%), followed by edema (31.3%), paresthesia (25%), hematoma (18.8%), post‐inflammatory hyperpigmentation (12.5%), and pruritus (12.5%). All reported effects were mild and transient, with resolution over the following days (Table 5).

**TABLE 5:** Frequency and distribution of adverse effects reported after microneedling radiofrequency treatment.

Variables	*n* = 16
**Presence of adverse effects—*n* (%)**	
Yes	12 (75.0)
No	4 (25.0)
**Erythema**	
Edema	10 (62.5)
Paresthesia	5 (31.3)
Hematoma	4 (25.0)
Erythema	3 (18.8)
Post‐inflammatory hyperpigmentation (PIH)	2 (12.5)
Pruritus	2 (12.5)
Paralysis	0 (0.0)
Burn	0 (0.0)
Scar	0 (0.0)
Blisters	0 (0.0)

### 3.8 PASMFRS (Physician‐Assisted Submental Fat Rating Scale)

One month after completion of the interventions and dermatologic ultrasound assessments, standardized photographic documentation was obtained. Image comparisons were performed by an external evaluator who analyzed pre‐ and post‐treatment photographs (Figures 2, 3, 4, 5). The PASMFRS, which classifies the degree of submental fullness, showed a statistically significant improvement (*p* = 0.001).

**FIGURE 2:**
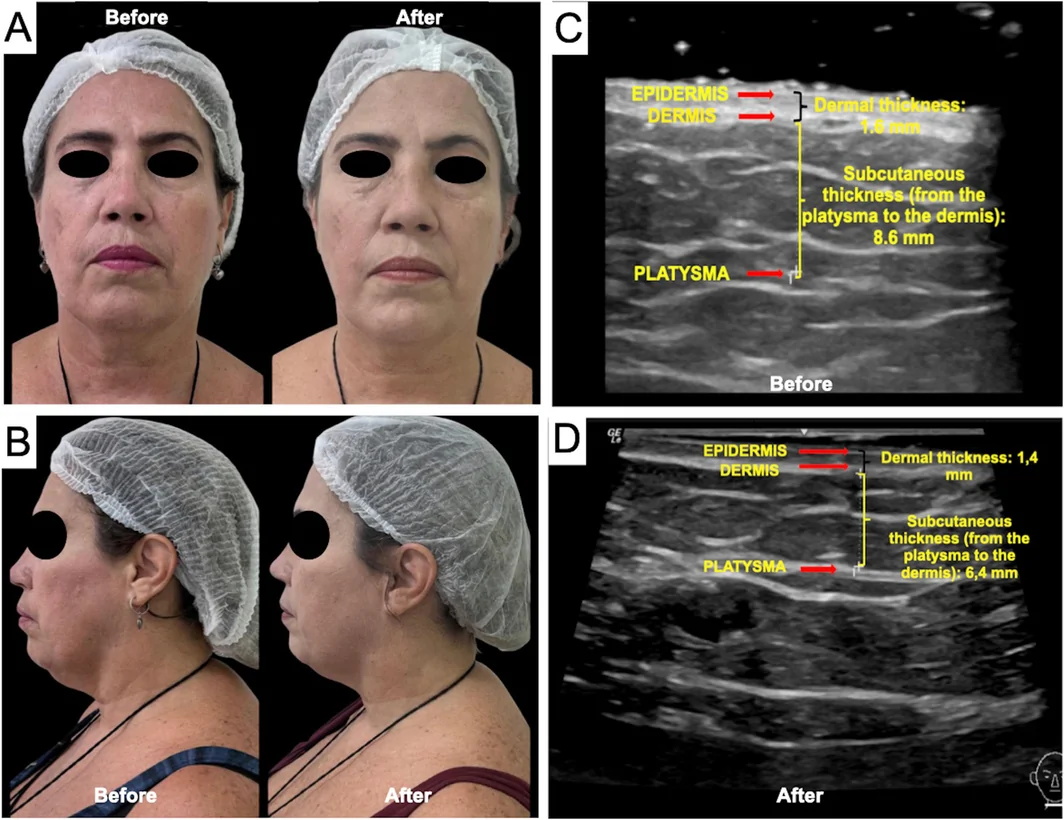
Photographic documentation of patient 1 before and after three sessions of multilayer microneedling radiofrequency (A, B). Dermatologic ultrasound using an 18‐MHz probe, comparing subcutaneous thickness (from the platysma to the dermis) before (C) and after three sessions of multilayer microneedling radiofrequency (D).

**FIGURE 3:**
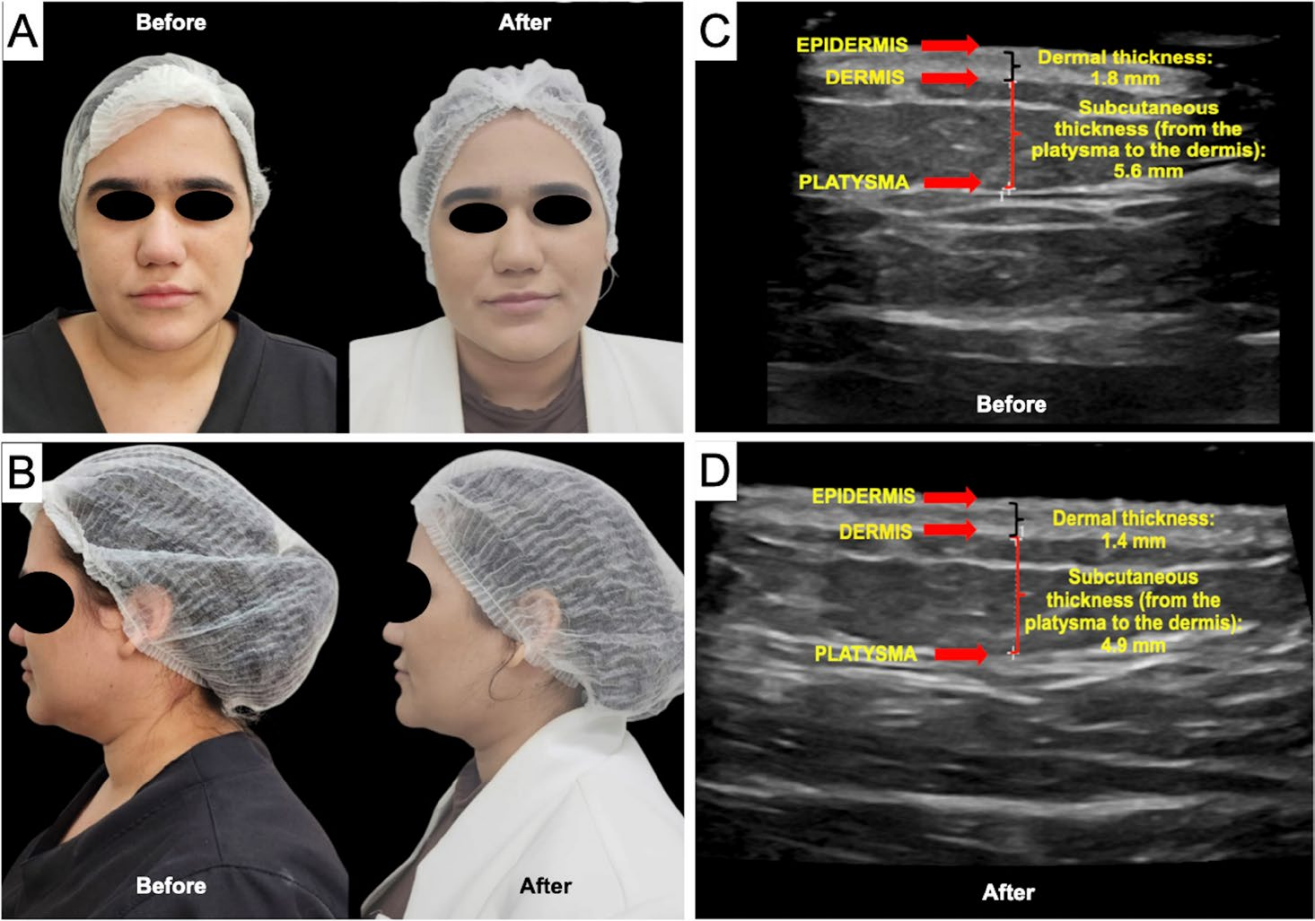
Photographic documentation of patient 2 before and after three sessions of multilayer microneedling radiofrequency (A, B). Dermatologic ultrasound using an 18‐MHz probe, comparing subcutaneous thickness (from the platysma to the dermis) before (C) and after three sessions of multilayer microneedling radiofrequency (D).

**FIGURE 4:**
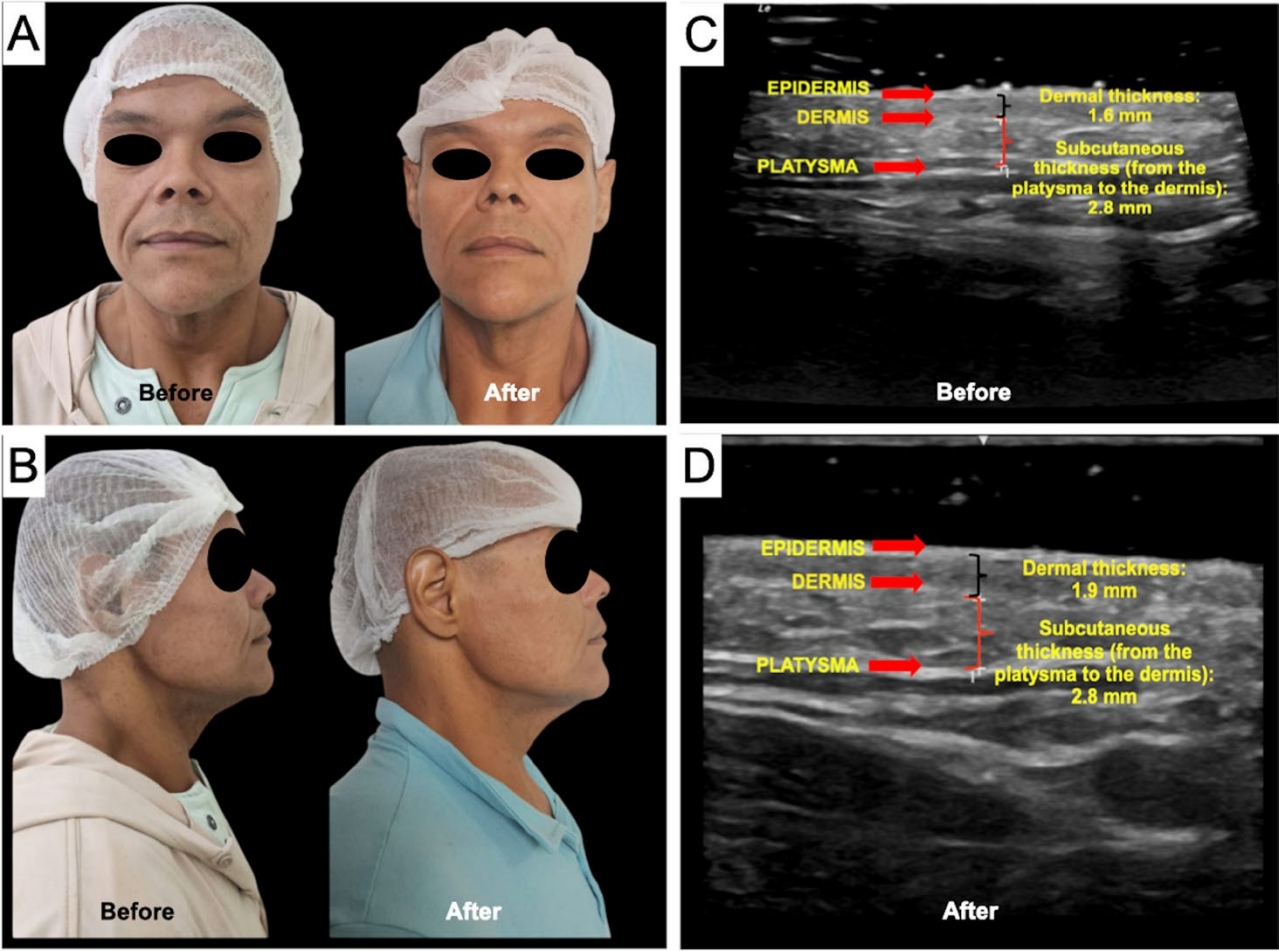
Photographic documentation of patient 3 before and after three sessions of multilayer microneedling radiofrequency (A, B). Dermatologic ultrasound using an 18‐MHz probe, comparing subcutaneous thickness (from the platysma to the dermis) before (C) and after three sessions of multilayer microneedling radiofrequency (D).

**FIGURE 5:**
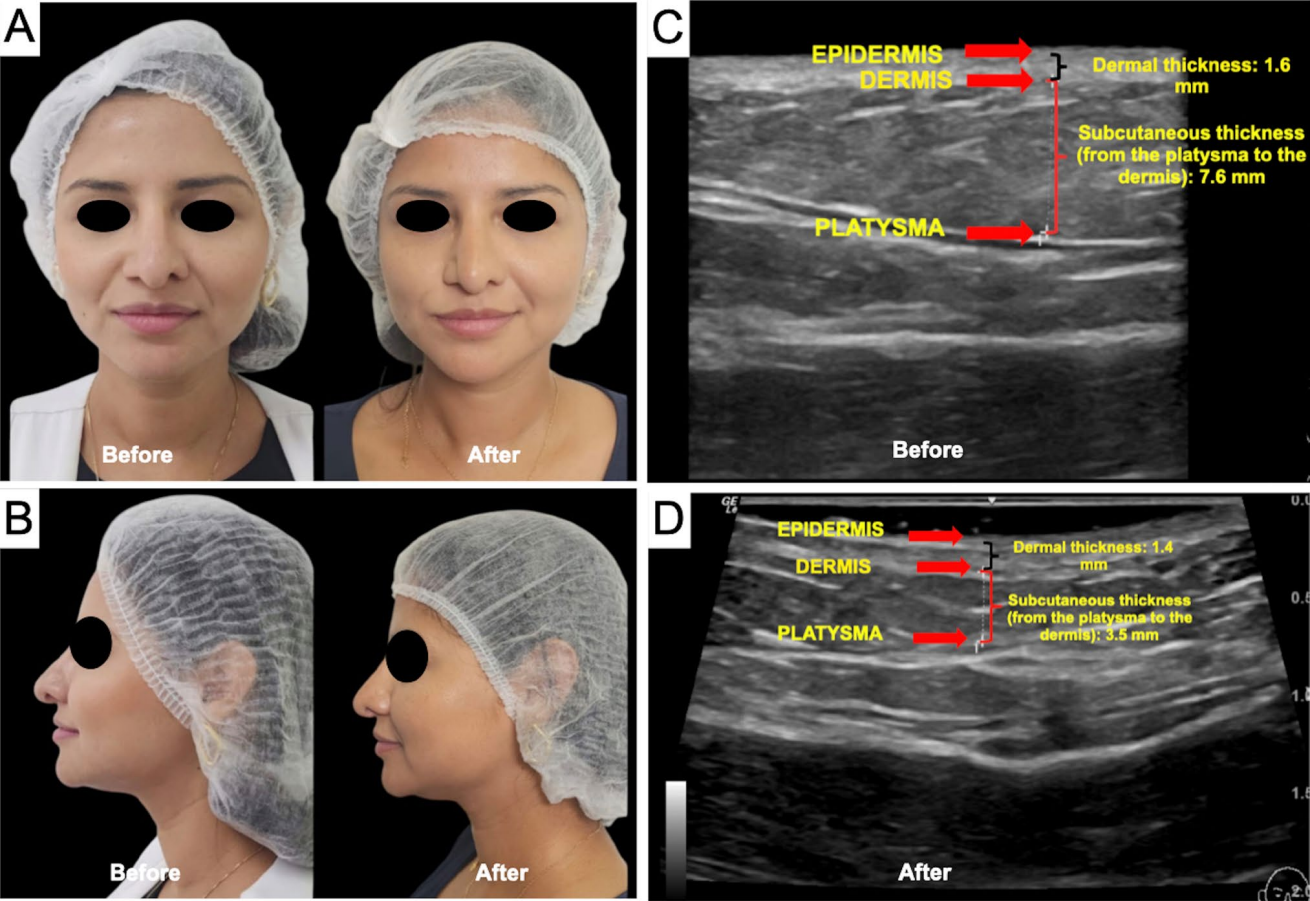
Photographic documentation of patient 4 before and after three sessions of multilayer microneedling radiofrequency (A, B). Dermatologic ultrasound using an 18‐MHz probe, comparing subcutaneous thickness (from the platysma to the dermis) before (C) and after three sessions of multilayer microneedling radiofrequency (D).

Of the 16 participants, 11 (68.75%) demonstrated improvement on the PASMFRS: one participant showed a two‐grade reduction, and 10 participants showed a one‐grade reduction. In contrast, 31.25% (5 participants) showed no improvement, maintaining the same baseline PASMFRS grade prior to the intervention (Table 6).

**TABLE 6:** Number and percentage of patients showing clinical improvement according to the Physician‐Assisted Submental Fat Rating Scale (PASMFRS).

Variables	*n* = 16
**SCALA PASMFRS—*n* (%)**	
Improvement	11 (68.75)
No difference	5 (31.25)

## 4 Discussion

In recent years, cutaneous aging has been one of the most extensively studied areas in cosmiatry and laser‐based therapies, particularly with regard to the face and neck. Growing interest in this field has driven the development of aesthetic devices, technologies, and products aimed at replacing surgical procedures or delaying invasive interventions as much as possible. The primary goals of these technologies are to minimize or eliminate downtime, reduce adverse effects, and simultaneously provide effective and satisfactory outcomes for patients [7].

Microneedling radiofrequency (MRF) has gained popularity since the early 2000s, with an estimated annual increase of 10% in demand for the procedure [7, 13]. This technology is widely used to treat age‐related skin laxity and localized fat in the anterior cervical region, promoting a more youthful and harmonious appearance. Its mechanism of action is based on controlled heating of dermal tissue to approximately 42°C, stimulating neocollagenesis and elastin formation [13, 14, 15]. MRF employs microneedles that deliver radiofrequency energy at variable depths and programmable energy levels, thereby promoting remodeling of the skin layers [11, 15, 16]. The present results demonstrated statistically significant effects in both dermatologic ultrasound parameters and participant satisfaction, with reported improvement in facial contour and reduction of submental fat. Among the 16 participants, 75% were women and 25% were men, with a mean age of 39.2 years. These findings reflect a predominance of female patients seeking aesthetic treatments, while also highlighting a growing interest among men. Additionally, an increasing number of younger patients are pursuing aesthetic interventions as a preventive strategy against early signs of aging, in contrast to previous trends in which such procedures were sought at more advanced ages.

In this study, the Megaderme Duo platform was used, and after three treatment sessions, statistically significant reductions were observed in subcutaneous tissue thickness (*p* = 0.001) and subplatysmal fat thickness (*p* = 0.034). Furthermore, in the mandibular angles, subcutaneous thickness decreased significantly on both sides (*p* < 0.001). These findings are consistent with the high level of participant satisfaction, with 93.8% reporting favorable outcomes.

A similar prospective study involving 24 adults treated with MRF for submental fat reduction demonstrated that scores on the Physician‐Assisted Submental Fat Rating Scale (PASMFRS) decreased significantly after 1 month, with even greater improvement after 2 months (*p* < 0.001). The mean submental fat volume also decreased significantly, from 20.44 ± 5.53 cc to 16.41 ± 4.58 cc (*p* < 0.001) [17]. These data corroborate the findings of the present study, in which comparative analysis of pre‐ and post‐treatment photographs using the PASMFRS revealed a statistically significant improvement (*p* = 0.001), with 68.75% of participants showing a reduction of at least one grade on the scale. Collectively, these results reinforce both the clinical and statistical efficacy of the treatment.

Regarding pain experienced during the sessions, the mean intensity was moderate despite the analgesic measures implemented to improve tolerability. Ketorolac tromethamine, an analgesic indicated for moderate to severe pain, was administered 1 h before the sessions and again 15 min prior to the procedure. In addition, a topical anesthetic containing 4% lidocaine was applied to the treated areas. Previous studies [18, 19] reported a mean pain score of 5.61 ± 1.91 on the VAS after three monthly treatments, which is comparable to the values observed in the present study. Other investigations have employed additional pain management strategies, such as compounded topical anesthetics with 23% lidocaine and 7% tetracaine, cold air cooling during the procedure, and even nitrous oxide sedation therapy to reduce patient anxiety [18, 19].

The reported side effects were mild and transient, with the most common being erythema (62.5%), edema (31.3%), paresthesia (25%), hematoma (18.8%), post‐inflammatory hyperpigmentation (12.5%), and pruritus (12.5%). No patients experienced severe or difficult‐to‐manage adverse effects, underscoring the safety of MRF for facial contouring and submental fat reduction, in agreement with the scientific literature. Previous studies similarly reported no long‐term adverse cutaneous reactions [17]. However, studies identified less common side effects, such as prolonged edema lasting more than 6 weeks (4.8%), skin induration persisting for more than 12 weeks (3.2%), and neuropraxia of the marginal mandibular branch (1.2%). It is noteworthy that neuropraxia is generally reversible, as it does not involve structural nerve damage but rather a transient alteration in nerve impulse conduction, which may result in temporary weakness or asymmetry of lower lip movements [20, 21, 22, 23].

## 5 Conclusion

Overall, multilayer microneedling radiofrequency using the Megaderme Duo LMG platform proved to be effective in reducing submental fat and improving facial contour. Moreover, it demonstrated a favorable safety profile, as evidenced by high levels of participant satisfaction and a low incidence of clinically significant adverse effects. Further studies involving larger patient populations are recommended to reinforce the robustness of these findings.

## Author Contributions

All authors contributed to the study conception, data collection, analysis, and manuscript preparation. All authors reviewed and approved the final version of the manuscript.

## Ethics Statement

This study was conducted in accordance with the principles of the Declaration of Helsinki. The present study complied with all requirements of Resolution No. 466, dated December 12, 2012, which establishes guidelines and regulatory standards for research involving human subjects, and Resolution No. 510, dated April 7, 2016, which addresses standards applicable to research in the Human and Social Sciences.

## Consent

All individuals who expressed interest in participating, met the inclusion criteria, and agreed to participate freely and voluntarily signed the Informed Consent Form (ICF) and the Authorization for Use of Data and Images.

## Conflicts of Interest

The authors declare no conflicts of interest.

## Data Availability

The data that support the findings of this study are available from the corresponding author upon reasonable request.
